# Clinical implication of tissue carcinoembryonic antigen expression in association with serum carcinoembryonic antigen in colorectal cancer

**DOI:** 10.1038/s41598-023-34855-9

**Published:** 2023-05-10

**Authors:** Abdulmohsin Fawzi Aldilaijan, Young Il Kim, Chan Wook Kim, Yong Sik Yoon, In Ja Park, Seok-Byung Lim, Jihun Kim, Jun-Soo Ro, Jin Cheon Kim

**Affiliations:** 1 Division of Colon and Rectal Surgery, Department of Surgery, Asan Medical Center, University of Ulsan College of Medicine, 88, Olympic-Ro 43-Gil, Songpa-Gu, Seoul, 05505 Republic of Korea; 2 Department of Pathology, Institute of Innovative Cancer Research, Asan Medical Center, University of Ulsan College of Medicine, Seoul, Korea; 3 Biomedical Research Institute, Seoul National University Hospital, Seoul, Republic of Korea

**Keywords:** Cancer, Gastrointestinal cancer

## Abstract

This study aimed to evaluate the prognostic significance of carcinoembryonic antigen (CEA) expression in tumor tissues of patients with colorectal cancer (CRC). The cohort included 7,412 patients with CRC from January 2010 to December 2015. Survival outcomes were assessed based on tissue CEA (t-CEA) patterns and intensities. Three-year (76.7% versus 81.3%) and 5-year (71.7% versus 77.6%, p < 0.001) disease-free survival (DFS) rates were significantly (p < 0.001) poorer in patients with a diffuse-cytoplasmic pattern than an apicoluminal pattern. Three-year (79% versus 86.6%) and 5-year (74.6% versus 84.7%) DFS rates were also significantly (p < 0.001) poorer in patients with high than low t-CEA intensity. Three-year (84.6% versus 88.4%) and 5-year (77.3% versus 82.6%) overall survival (OS) rates were significantly (p < 0.001) poorer in patients with diffuse-cytoplasmic than apicoluminal pattern of CEA expression, and both 3-year (86.7% versus 91.2%) and 5-year (80.1% versus 87.7%) OS rates were significantly (p < 0.001) poorer in patients with high than low t-CEA intensity. Multivariate analyses showed that high-intensity t-CEA was independently associated with DFS (p = 0.02; hazard ratio [HR] = 1.233) and OS (p = 0.032; HR = 1.228). Therefore, high-intensity t-CEA is a significant prognostic factor in CRC, independent of serum CEA (s-CEA), and can complement s-CEA in predicting survival outcomes after CRC resection.

## Introduction

Colorectal cancer (CRC) is the third most common malignancy worldwide and the second most frequent cause of cancer-related deaths^1^. In the Republic of Korea (South Korea), CRC is the fourth most common malignancy and the third leading cause of cancer-related deaths^2, 3^. Despite advances in medical and surgical management, patient survival continues to be reduced by disease recurrence. Prognosis and overall survival (OS) in CRC have been found to correlate with the TNM staging system currently used by the American Joint Committee on Cancer (AJCC) and the International Union Against Cancer (UICC)^4^. Adverse features predictive of recurrence and poorer outcome after curative operation include tumor differentiation, lymphovascular invasion (LVI), perineural invasion (PNI), margin status, and mismatch repair protein status^5–8^.

Serum carcinoembryonic antigen (s-CEA) is a glycoprotein present in the human digestive system, elevated in patients with colon and rectal neoplasms^9^. This protein is encoded by CEACAM5 and expressed in colorectal epithelial cells, where it functions in cell recognition and intercellular adhesion^10–12^. In CRC, CEA expressed following the disruption of normal tissue structure and the loss of polarization of neoplastic cells is secreted into the blood stream, eventually resulting in an increase in s-CEA concentration^13^. Although s-CEA level is neither sufficiently sensitive nor specific as a screening tool for CRC, it plays an important role in surveillance after surgical resection^14^. In addition, s-CEA can be targeted in cancer imaging and active immunotherapy^15^.

Elevated preoperative s-CEA concentration, defined as > 5 ng/ml or more than two-fold higher than the normal cut-off value, is significantly associated with poorer overall and higher cancer-specific mortality in CRC patients^16–21^. Because preoperative s-CEA concentration > 5 ng/ml is an independent prognostic factor for poor OS, chemotherapy or intensive follow-up strategies should be considered, particularly in patients with negative lymph node metastasis, if preoperative s-CEA level is > 10 ng/ml^8, 19, 22, 23^. Recently, s-CEA has been targeted in tumor imaging, using recombinant vaccinia CEA (rV-CEA), or for active immunotherapy with recombinant adenovirus 5 (CEA/MUC1/Brachyury)^24^.

In addition to measuring preoperative s-CEA concentration, the expression of tissue CEA (t-CEA) can be immunohistochemically assessed in colorectal mucosa and tumor tissues. t-CEA is rarely expressed in normal colorectal mucosa but is consistently found in colorectal neoplasms, with different expression patterns and intensities^15, 25^. t-CEA expression patterns have been described as apicoluminal (AL), diffuse-cytoplasmic (DC), or a combination of the two. The DC pattern and high levels of expression have been associated with tumor aggressiveness, including LVI^26^. Studies have suggested that the DC pattern is associated with higher preoperative s-CEA levels, higher rates of lymph node and liver metastases, and higher recurrence and lower survival rates than the AL pattern^26–29^, although contradictory findings have also been reported^30^.

Few studies to date have assessed the associations of t-CEA expression pattern and intensity with long-term survival outcomes in patients with CRC. The present study evaluated the relationships between t-CEA expression and long-term survival in patients with CRC.

## Results

### Clinicopathological characteristics of patients and tumors

During the study period (January 2010 to December 2015), 10,566 patients underwent colorectal resection at Asan Medical Center. The 7412 included patients consisted of 4343 (58.6%) men and 3069 (41.4%) women, of mean age 61.7 ± 11.4 years. Of these patients, 5092 (68.7%) were diagnosed with colon cancer and 2320 (31.3%) with rectal cancer. Preoperative s-CEA level was high (> 6 mg/ml) in 1690 (23.2%) patients. Advanced tumor stages (III and IV) were found in 3505 (47.3%) patients. Most patients (6853, 92.5%) had well- and moderately differentiated adenocarcinoma, whereas 556 (7.5%) had unfavorable differentiation. LVI and PNI were identified in 2695 (36.4%) and 1855 (25.1%) patients, respectively.

### Tissue carcinoembryonic antigen expression

Immunohistochemical analysis of t-CEA expression showed that resected tumor tissue of 5004 (67.5%) patients had the AL pattern and 2408 (32.5%) had the DC pattern. The DC pattern was significantly associated (p < 0.001) with factors associated with poor patient prognosis, including elevated preoperative s-CEA concentration, advanced TNM stage, unfavorable tumor histology, and LVI/PNI. High-intensity t-CEA expression was observed in tumor samples from 6629 (89.4%) patients and low intensity expression in 783 (10.6%) patients. High-intensity expression correlated significantly with elevated preoperative s-CEA concentration, advanced TNM stage, and LVI/PNI (p < 0.001, Table 1).

**Table 1:** Clinicopathological characteristics of the study patients stratified by tissue CEA expression pattern and density.

Variable	CEA pattern			CEA intensity		
	Apicoluminal (*n* = 5004)	Diffuse cytoplasmic (*n* = 2408)	*p*-value	Low ( +) (*n* = 783)	High (+ + / + + +) (*n* = 6629)	*p*-value
Sex						
Female	2108 (42.1%)	961 (39.9%)	0.069	324 (41.4%)	2745 (41.4%)	0.987
Male	2896 (57.9%)	1447 (60.1%)		459 (58.6%)	3884 (58.6%)	
Age (year)						
< 60	2153 (43.0%)	962 (40.0%)	0.012	340 (43.4%)	3395 (51.2%)	0.403
> = 60	2851 (57.0%)	1446 (60.0%)		443 (56.6%)	3854 (58.1%)	
Tumor location						
Colon	3459 (69.1%)	1633 (67.8%)	0.255	603 (77.0%)	4489 (67.7%)	< 0.001
Rectum	1545 (30.9%)	775 (32.2%)		180 (23.0%)	2140 (32.3%)	
Pre-op s-CEA						
≤ 6	3961 (80.6%)	1637 (69.0%)	< 0.001	643 (86.5%)	4955 (75.7%)	< 0.001
> 6	956 (19.4%)	734 (31.0%)		100 (13.5%)	1590 (24.3%)	
pT category						
pT0-3	4412 (88.2%)	2027 (84.2%)	< 0.001	704 (89.9%)	5735 (86.5%)	0.008
pT4	592 (11.8%)	1947 (15.8%)		79 (10.1%)	894 (13.5%)	
pN category						
pN0	2963 (59.2%)	1192 (49.5%)	< 0.001	524 (66.9%)	3631 (54.8%)	< 0.001
pN +	2401 (40.8%)	1216 (50.5%)		259 (33.1%)	2998 (45.2%)	
Stage category						
0-I-II	2801 (56.0%)	1106 (45.9%)	< 0.001	507 (64.8%)	3400 (51.3%)	< 0.001
III-IV	2203 (44.0%)	1302 (54.1%)		276 (35.2%)	3229 (48.7%)	
LVI						
Absent	3318 (66.4%)	1392 (57.8%)	< 0.001	593 (75.7%)	4117 (62.2%)	< 0.001
Present	1680 (33.6%)	1015 (42.2%)		190 (24.3%)	250 (37.8%)	
PNI						
Absent	3882 (77.7%)	1659 (69.0%)	< 0.001	666 (85.2%)	4875 (73.7%)	< 0.001
Present	1111 (22.3%)	744 (31.0%)		116 (14.8%)	1739 (26.3%)	
Differentiation						
WD/MD	4669 (93.3%)	2184 (90.8%)	< 0.001	694 (88.6%)	6159 (93.0%)	< 0.001
PD/MUC/SRC	334 (6.7%)	222 (9.2%)		89 (11.4%)	467 (7.0%)	

*s-CEA* serum carcinoembryonic antigen, *WD* well-differentiated, *MD* moderately differentiated, *PD* poorly differentiated, *MUC* mucinous, *SRC* signet ring cell, *LVI* lymphovascular invasion, *PNI* perineural invasion.

### Recurrence and survival outcomes

After a mean follow-up time of 86.1 ± 33.0 months, 985 (13.3%) patients experienced tumor recurrence, including 74 (7.5%) with locoregional and 839 (85.2%) with systemic recurrences. Of the 839 patients who experienced systemic recurrence, 547 (65.2%) experienced recurrences to the liver, lungs, and/or distant nodes. Other systemic recurrences included peritoneal metastasis in 109 (13.0%) patients; recurrences to other organs, including the ovaries, brain, bones, adrenal glands, and spleen in 28 (3.4%); and multiple routes in 85 (10.1%).

Overall recurrence rates were significantly greater in patients with the DC than the AL pattern (p = 0.001) and in patients with high than low t-CEA expression intensity (p < 0.001). In patients with systemic (distant) recurrences, the DC pattern tended to show greater peritoneal metastasis than the AL pattern (15.9% versus 11.3%; Table 2).

**Table 2:** Recurrence of the study patients stratified by tissue CEA expression pattern and density.

Variable	CEA pattern			CEA intensity		
	Apicoluminal (*n* = 5004)	Diffuse cytoplasmic (*n* = 2408)	*p*-value	Low ( +) (*n* = 783)	High (+ + / + + +) (*n* = 6629)	*p*-value
Recurrence						
No	4389 (87.6%)	2041 (84.8%)	0.001	718 (91.7%)	5709 (86.1%)	< 0.001
Yes	618 (12.4%)	367 (15.2%)		65 (8.3%)	920 (13.9%)	
Recurrence route category						
Locoregional	45 (7.8%)	29 (8.6%)	0.702	6 (10.5%)	68 (7.9%)	0.489
Systemic	529 (92.2%)	310 (91.4%)		51 (89.5%)	788 (92.1%)	
Recurrence route						
Locoregional	45 (7.8%)	29 (8.6%)	0.328	6 (10.5%)	68 (7.9%)	0.960
Hemato-lymphatogenous (Liver/Lung/Distant LN)	384 (66.9%)	211 (62.2%)		35 (61.4%)	560 (65.4%)	
Bone/Brain/Others	21 (3.7%)	10 (2.9%)		2 (3.5%)	29 (3.4%)	
Multivisceral	59 (10.3%)	35 (10.3%)		6 (10.5%)	88 (10.3%)	
Peritoneal metastasis	65 (11.3%)	54 (15.9%)		8 (14.0%)	111 (13.0%)	

*CEA* carcinoembryonic antigen, *LN* lymph node.

**Figure 1:**
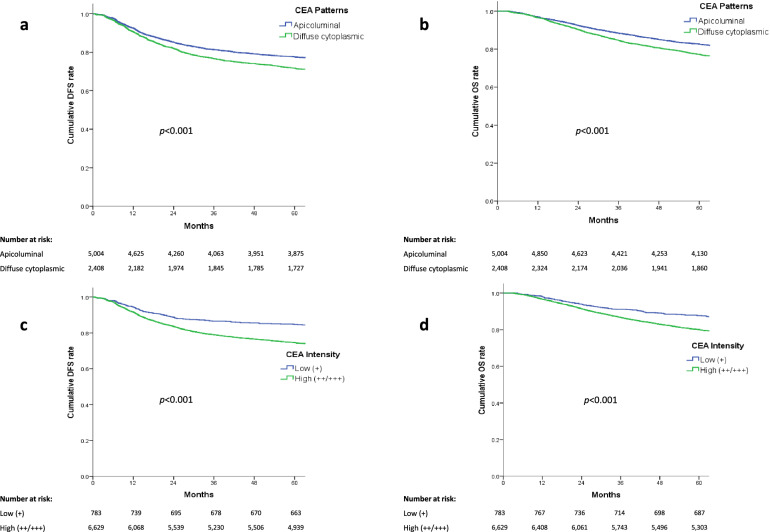
Kaplan–Meier analyses comparing (**a,c**) disease-free survival and (**b,d**) overall survival in patients with (**a,b**) the apicoluminal and diffuse-cytoplasmic t-CEA expression patterns, and (**c,d**) low and high-intensity t-CEA expression.

The mean ± SD DFS in the overall patient cohort was 77.5 ± 37.6 months, and the mean ± SD OS was 82.8 ± 33.0 months. Univariate analyses showed that 3- and 5-year DFS and OS rates differed significantly between groups of patients with different t-CEA patterns and intensities (p < 0.001; Fig. 1). For example, the 3- and 5-year DFS rates were 76.7% and 71.7%, respectively, in patients with the DC pattern, and 81.3% and 77.6%, respectively, in patients with the AL pattern. Similarly, the 3- and 5-year OS rates were 84.6% and 77.3%, respectively, in patients with the DC pattern, and 88.4% and 82.6%, respectively, in patients with the AL pattern. Survival rates were also significantly lower in patients with high than low intensity t-CEA expression. For example, the 3- and 5-year DFS rates were 79% and 74.6%, respectively, in patients with high-intensity expression, compared with 86.6% and 84.7%, respectively, in patients with low intensity expression. Similarly, the 3- and 5-year OS rates were 86.7% and 80.1%, respectively, in patients with high-intensity expression, compared with 91.2% and 87.7%, respectively, in patients with low intensity expression. Kaplan–Meier survival analysis according to each TNM stages was performed. In stage III patients, significant poor DFS and OS rate was shown in the high-intensity t-CEA expression patients (Supplementary Fig. 3). In stage II patients, high-intensity t-CEA was shown to have significant poor DFS but not OS rate (Supplementary Fig. 2). In stage 0 ~ I and IV, t-CEA did not show significant correlation with survival outcomes (Supplementary Figs. 1 and 4).

Multivariate analyses showed that high-intensity tissue CEA expression was independently associated with DFS (p = 0.02; HR = 1.233) and OS (p = 0.032; HR = 1.228). t-CEA expression pattern was not a prognostic factor in the multivariable analysis (Table 3).

**Table 3:** Multivariate analysis of prognostic factors of DFS and OS.

Variable	DFS			OS		
	Hazard ratio	95% CI	*p*-value	Hazard ratio	95% CI	*p*-value
Sex, male	1.153	1.054–1.261	0.002	1.138	1.034–1.253	0.008
Age (year) > 60	1.492	1.361–1.636	< 0.001	1.780	1.609–1.968	< 0.001
Location of tumor, rectum	1.012	0.921–1.113	0.803	0.999	0.902–1.105	0.981
Preoperative s-CEA > 6 ng/ml	2.108	1.922–2.312	< 0.001	2.132	1.932–2.353	< 0.001
Stage category (III, IV)	2.547	2.285–2.839	< 0.001	2.603	2.314–2.928	< 0.001
LVI present	1.466	1.329–1.617	< 0.001	1.548	1.393–1.719	< 0.001
PNI present	1.613	1.465–1.775	< 0.001	1.626	1.467–1.801	< 0.001
Differentiation (PD/MUC/SRC)	1.405	1.220–1.619	< 0.001	1.581	1.365–1.831	< 0.001
t-CEA expression pattern (DC)	0.953	0.869–1.045	0.302	0.960	0.870–1.059	0.417
t-CEA expression intensity (high)	1.233	1.033–1.471	0.020	1.228	1.017–1.483	0.032

*DFS* disease-free survival, *OS* Overall survival, *LVI* lymphovascular invasion, *PNI* perineural invasion, *PD* poorly differentiated, *MUC* mucinous, *SRC* signet ring cell, *DC* diffuse cytoplasmic.

**Figure 2:**
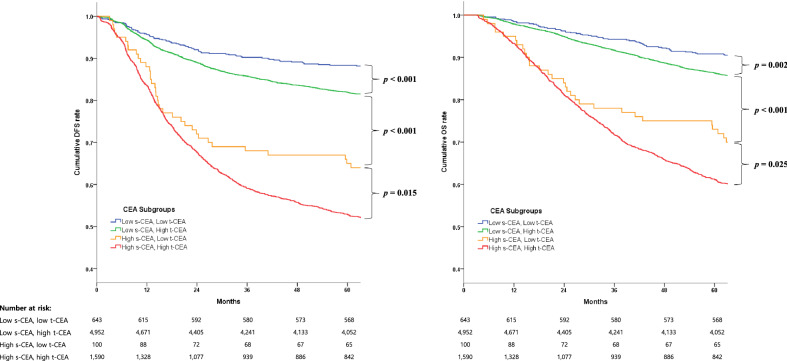
Kaplan–Meier analyses comparing disease-free survival and overall survival in patients with low preoperative s-CEA and low intensity t-CEA expression, low preoperative s-CEA level and high-intensity t-CEA expression, high preoperative s-CEA and low intensity t-CEA expression, and high preoperative s-CEA and high-intensity t-CEA expression.

Subgroup analyses were performed in patients assorted by both t-CEA expression intensity and preoperative s-CEA level. In patients with low preoperative s-CEA, those with high-intensity t-CEA expression had significantly poorer DFS (p < 0.001) and OS (p = 0.002) rates than patients with low intensity t-CEA expression. Similarly, among patients with high preoperative s-CEA, high-intensity t-CEA expression had significantly poorer DFS (p = 0.015) and OS (p = 0.025) rates than low intensity t-CEA expression (Fig. 2).

## Discussion

CEA acts as a metastatic potentiator through both homophilic and heterophilic binding^31^. CEA actively participates in the immune-related tumor microenvironment through a MHC class I-independent inhibitory pathway that mediates homophilic CEA interactions or heterophilic interactions of CEA with CEACAM1^24^. The expression of CEA, especially on the cell membrane, as in patients with the DC pattern, interferes with the signaling of DR5 by direct interaction through the PELPK sequence of the CEA hemophilic binding domain, reducing caspase-8 activity and anoikis^32^. This biological behavior of CEA, along with the close correlation of t-CEA expression with LVI/PNI, suggests that t-CEA expression may play a significant role in a pre-metastatic niche establishing a potential tumor microenvironment (TME). Curative surgical resection may therefore be the most efficient method of removing the primary tumor as well as the TME.

High preoperative s-CEA level is prognostic of poor survival in patients with CRC^16–19^. The present study found that t-CEA expression intensity and pattern correlated significantly with preoperative s-CEA level. Although many previous studies have reported a lack of correlation between preoperative s-CEA levels and t-CEA expression^29, 30, 33, 34^, one study confirmed this relationship^35^. These discrepancies may be due to differences in categorization of t-CEA expression patterns and intensities, and the small populations sizes (30–517 patients) in these studies.

Of the 7412 patients included in the present study, only 100 (1.3%) showed inverse relationships between t-CEA expression intensities and preoperative s-CEA levels. Low t-CEA expression intensity in patients with high preoperative s-CEA levels may be explained by factors unrelated to malignancy, including the wide range of normal preoperative s-CEA concentrations among healthy people, the effects of age and benign conditions, the high variability of liver metabolic rates, and the long half-life of glycoproteins. These findings may also be explained by the movement over time of CEA molecules from tissue to blood.

In agreement with previous studies, the present study showed that both high-intensity t-CEA expression and the DC pattern were significantly associated with higher tumor recurrence rates^29, 34^. Preoperative s-CEA concentration is also related to higher recurrence rates, as confirmed in the present study. The ability of t-CEA expression intensity to predict recurrence was especially noticeable among patients with low preoperative s-CEA levels, with patients having high-intensity t-CEA expression showing significantly higher rates of recurrence regardless of low preoperative s-CEA level.

This study showed that both elevated preoperative s-CEA and high-intensity t-CEA expression^28, 34^ were independently prognostic of poorer DFS. When divided into four subgroups based on both preoperative s-CEA level and t-CEA expression intensity, DFS was worse in groups with high-intensity t-CEA expression regardless of preoperative s-CEA levels. Taken together, these findings suggest that t-CEA expression intensity plays a complementary role as an adjunctive measurement of preoperative s-CEA level. The intensity of t-CEA expression may therefore be a reliable and accurate measure of patient prognosis beginning at an early stage of treatment.

Several ambiguous results from the present and previous studies require further explanation. Although Kaplan–Meier analysis showed that the DC pattern was associated with significantly poorer DFS and OS, these correlations were not statistically significant on multivariate analyses. Although the DC pattern is indicative of CEA distribution in the cytoplasm and may be associated with poorer prognosis, multivariable analysis showed that only high-intensity t-CEA expression was significantly associated with poorer survival outcomes. Also, unfavorable histologic differentiation was significantly associated with DC expression pattern, whereas high-intensity t-CEA expression was significantly associated with favorable tumor differentiation. A previous study also found that t-CEA expression intensity was higher in well differentiated than poorly differentiated colorectal adenocarcinomas^33^. Further research is needed to explain these phenomena.

This study is limited by its non-randomized design and the retrospective nature of the data. For example, the inability to measure t-CEA in patients with unresectable CRC required excluding this group of patients. The results of this study suggested that advanced CRC stages are associated with high intensity or the DC pattern of t-CEA expression. Some discrepancies with previous studies may be caused by differences in pathologic interpretations. For example, determination of t-CEA expression patterns is subjective, as these evaluations are related to the depth of CEA distribution. Regarding this issue, due to the substantially large number of patients included in this study, the reliability of t-CEA results from the pathologists could not be assessed. However, t-CEA expression pattern has been analyzed for over a decade in the present tertiary medical center and experience has accumulated to form a consensus, resulting in consistent pathologic reports.

High intensity of tissue carcinoembryonic antigen is a significant prognostic factor in patients with colorectal cancer, independent of serum carcinoembryonic antigen. Tissue carcinoembryonic antigen can be used complementary with serum carcinoembryonic antigen to predict survival outcomes after colorectal cancer resection.

## Methods

### Data collection and study design

**Figure 3:**
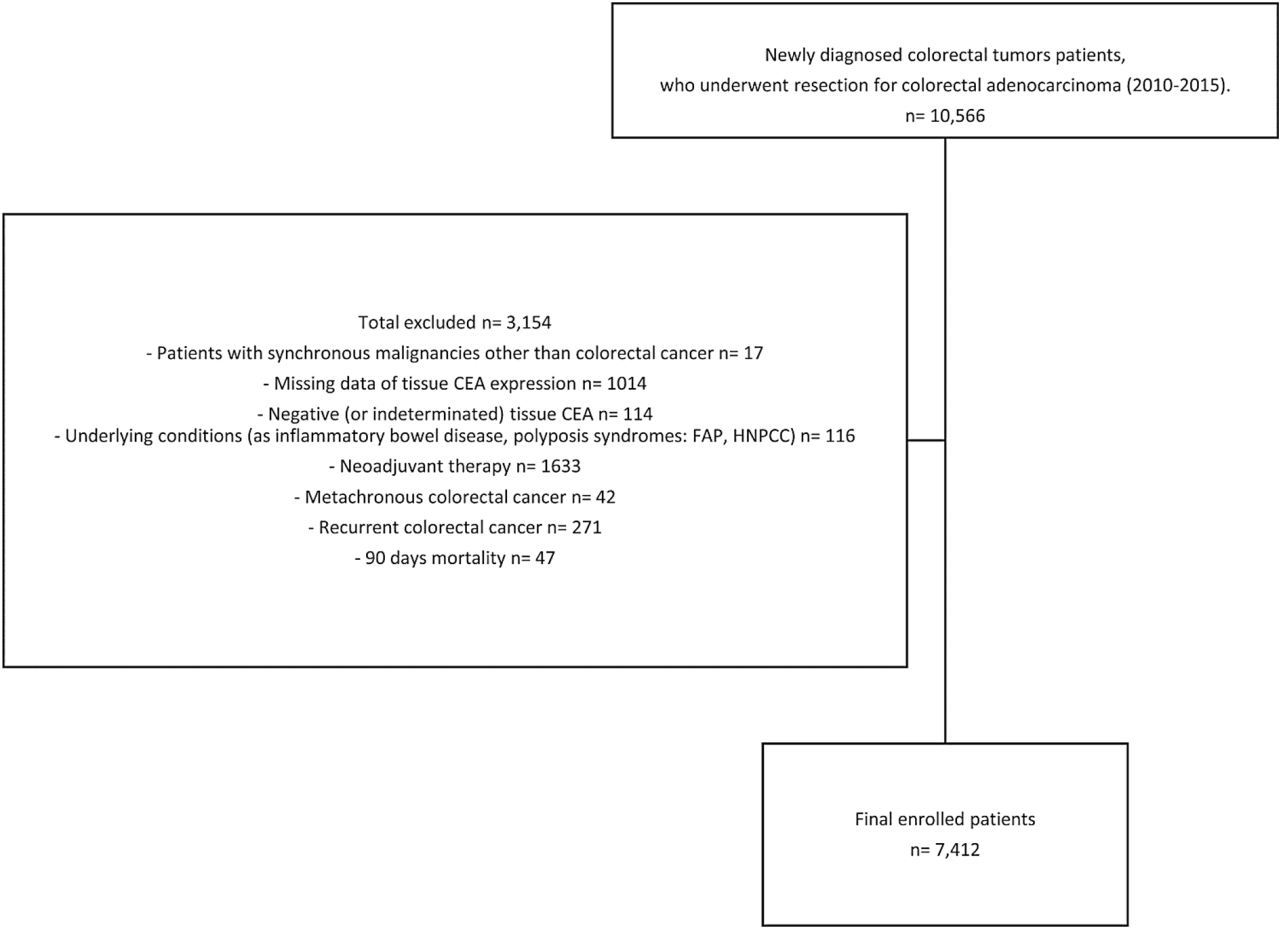
Flowchart of study population.

The medical records of 10,566 patients diagnosed with CRC who underwent resection between January 2010 and December 2015 at Asan Medical Center (Seoul, South Korea) were reviewed retrospectively. Patients with inflammatory bowel disease, familial adenomatous polyposis, and hereditary nonpolyposis colorectal cancer were excluded. Also excluded were patients with missing data on t-CEA expression, those who received neoadjuvant therapy, patients with synchronous malignancies other than CRC, patients with metachronous or recurrent CRC, and those who died within 90 days of resection. Patients with incomplete data due to loss to follow-up were excluded. Unresectable stage IV patients who received palliative resection of the primary tumor due to complications related to tumor (obstruction, bleeding, ischemia) were included. Thus, a total of 7412 patients was enrolled in this study (Fig. 3).

The study protocol was approved by the Institutional Review Board at Asan Medical Center (approval number: 2021–0908), which waived the requirement for informed consent due to the retrospective design of this study. This study was conducted according to the Declaration of Helsinki^36^.

### Evaluation of CEA

**Figure 4:**
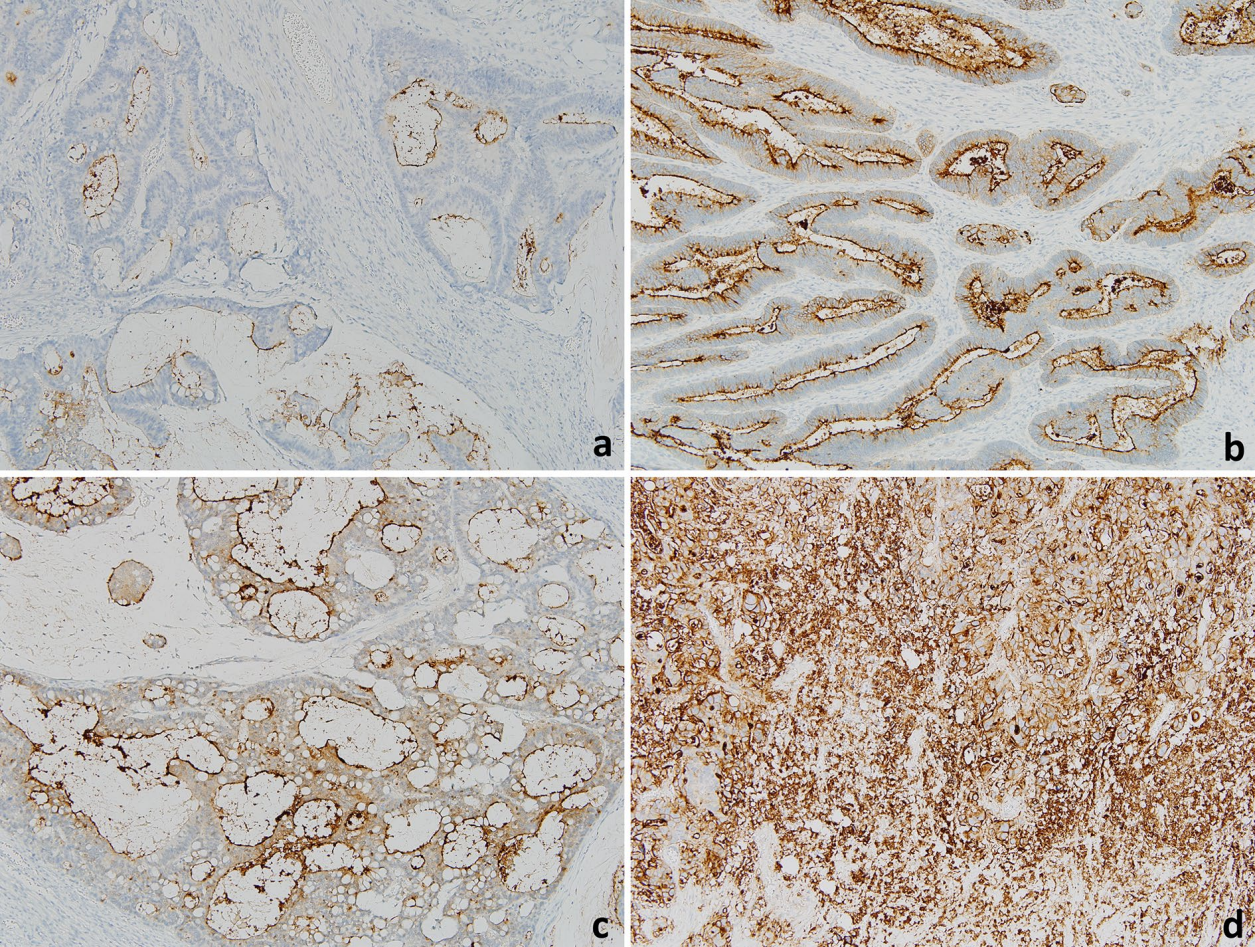
Carcinoembryonic antigen (CEA) distribution in colorectal cancer. (**a**) Apicoluminal pattern with low intensity; (**b**) Apicoluminal pattern with high intensity; (**c**) Diffuse-cytoplasmic pattern with low intensity; (**d**) Diffuse-cytoplasmic pattern with low intensity (CEA immune staining; × 100).

t-CEA distribution in CRC tissue was assessed immunohistochemically, using 1:1600 mouse monoclonal, clone CEA31, catalog No.236 M-96, CELL MARQUE, CALIFONIA, USA. The distribution patterns in neoplastic tissue were categorized into two patterns (Fig. 4), with the AL pattern defined as CEA immunoreactivity along the cytoplasmic membrane and the DC pattern as homogeneous staining within the cytoplasm including the cytoplasmic membrane. Samples showing both the AL and DC patterns were categorized as DC. The intensity of CEA immune staining was classified as weak, moderate, or strong, depending on the proportion of tumor cells presenting more than moderate staining intensity (i.e. < 25%, 25–50%, or > 50%, respectively). Staining was performed in one whole slide section and intratumoral heterogeneity was not shown in most cases. Weak staining was scored as low intensity, whereas moderate and strong were scored as high intensity. Preoperative s-CEA concentrations > 6 ng/ml were defined as high according to the criteria of Asan Medical Center, in which routine modality of detecting s-CEA is by radioimmunoassay (RAI).

### Statistical analysis

Primary outcomes were disease-free survival (DFS) and OS. DFS was defined as the interval between the date of surgery and the date of cancer recurrence or death from any cause. OS was defined as the interval between the date of surgery to the date of death from any cause or the end of the study. Categorical variables were analyzed using chi-square tests. Continuous variables were expressed as mean ± standard deviation and compared using Student’s t-tests. Survival was analyzed using the Kaplan–Meier method and compared by log-rank test. Confounding factors, including sex, age, preoperative s-CEA concentration, tumor location (colon *versus* rectum), tumor differentiation, LVI, PNI, and tumor stage, were adjusted using multivariable analysis (Cox proportional hazards model). All statistical analyses were performed using IBM SPSS^®^ version 26.0 (IBM, Armonk, NY), with p < 0.05 considered statistically significant.

## Supplementary Information

Supplementary Figure 1.

Supplementary Figure 2.

Supplementary Figure 3.

Supplementary Figure 4.

Supplementary Legends.

## Data Availability

The data that support the findings of this study are available from the corresponding author on request.
