# Supplementary material for: Clinical implication of tissue carcinoembryonic antigen expression in association with serum carcinoembryonic antigen in colorectal cancer
Source: Sci Rep. 2023 May 10;13:7616. doi: 10.1038/s41598-023-34855-9 (PMC10172318; doi:10.1038/s41598-023-34855-9)
Supplement: Supplementary file 1 — Supplementary Figure 1. [file 41598_2023_34855_MOESM1_ESM.pdf]

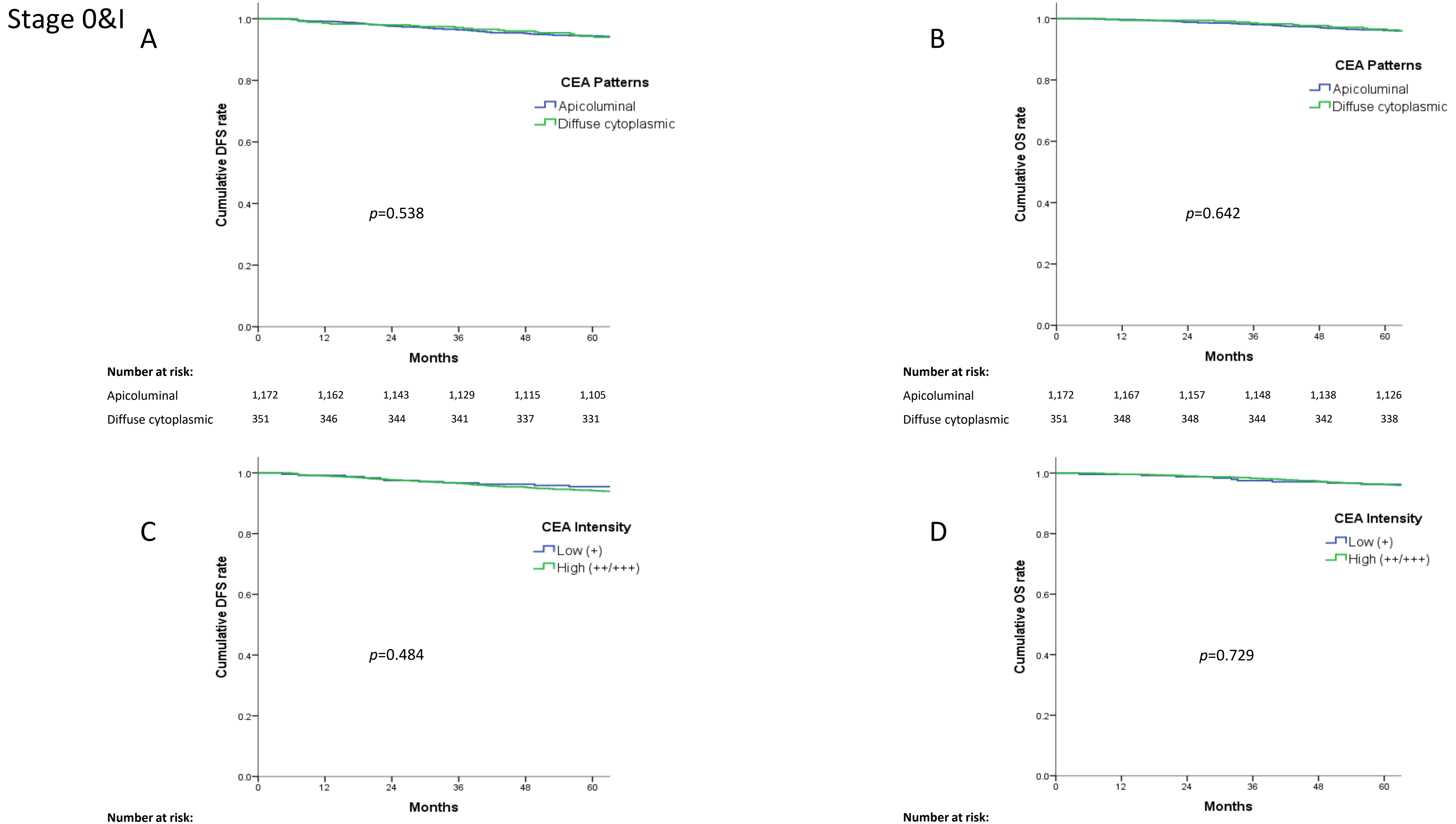

A — Number at risk:

	0	12	24	36	48	60
Apicoluminal	1,172	1,162	1,143	1,129	1,115	1,105
Diffuse cytoplasmic	351	346	344	341	337	331

B — Number at risk:

	0	12	24	36	48	60
Apicoluminal	1,172	1,167	1,157	1,148	1,138	1,126
Diffuse cytoplasmic	351	348	348	344	342	338

C — Number at risk:

	0	12	24	36	48	60
Low (+)	242	240	236	234	233	231
High (++/+++)	1,281	1,268	1,251	1,236	1,219	1,205

D — Number at risk:

	0	12	24	36	48	60
Low (+)	242	241	239	236	235	233
High (++/+++)	1,281	1,274	1,266	1,257	1,245	1,231
