# Supplementary material for: Clinical implication of tissue carcinoembryonic antigen expression in association with serum carcinoembryonic antigen in colorectal cancer
Source: Sci Rep. 2023 May 10;13:7616. doi: 10.1038/s41598-023-34855-9 (PMC10172318; doi:10.1038/s41598-023-34855-9)
Supplement: Supplementary file 2 — Supplementary Figure 2. [file 41598_2023_34855_MOESM2_ESM.pdf]

Stage II

A

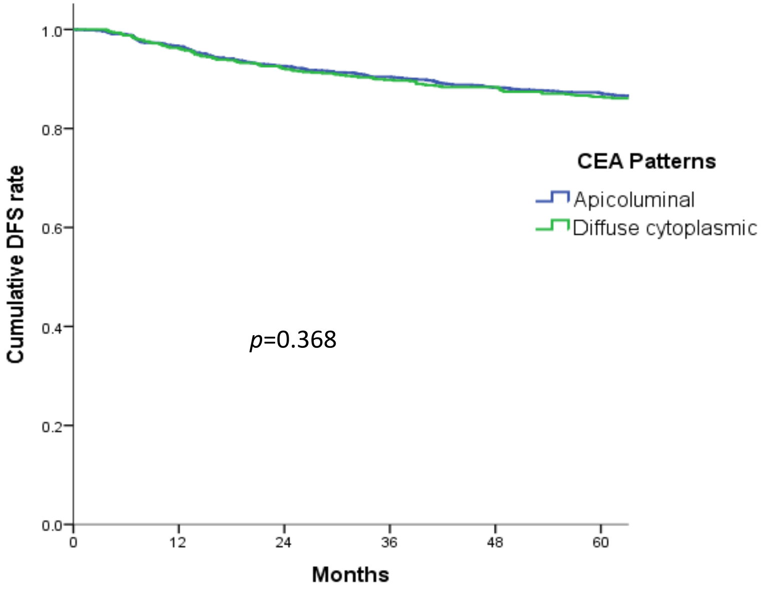

Number at risk:

Apicoluminal	1,629	1,573	1,509	1,471	1,435	1,417
Diffuse cytoplasmic	756	727	696	679	668	653

B

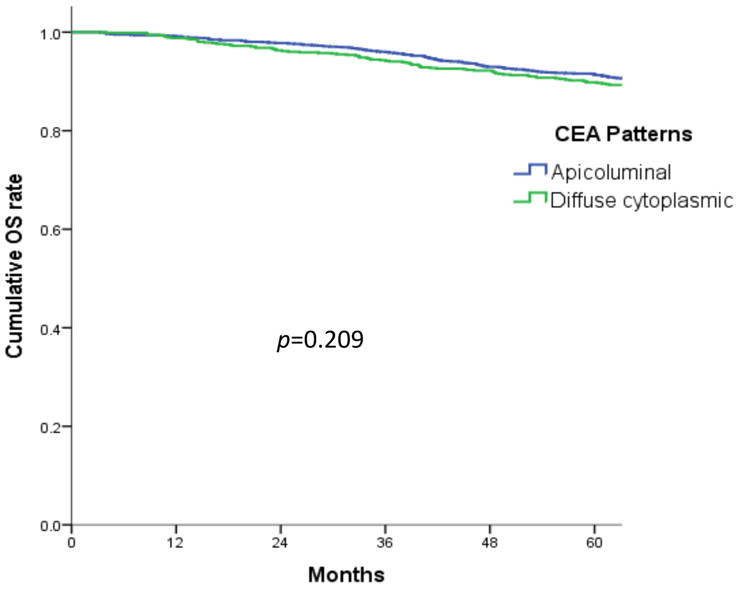

Number at risk:

Apicoluminal	1,628	1,614	1,592	1,563	1,513	1,488
Diffuse cytoplasmic	756	747	728	713	697	679

C

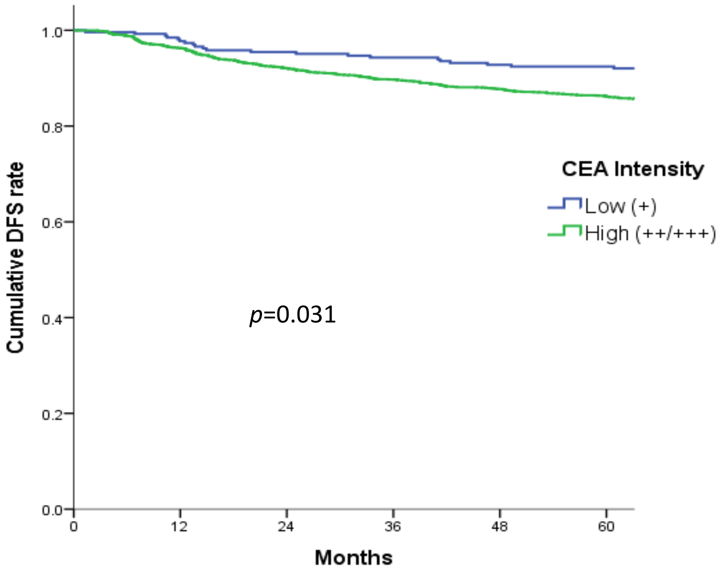

Number at risk:

Low (+)	264	258	252	249	245	244
High (++/+++)	2,121	2,042	1,953	1,901	1,858	1,825

D

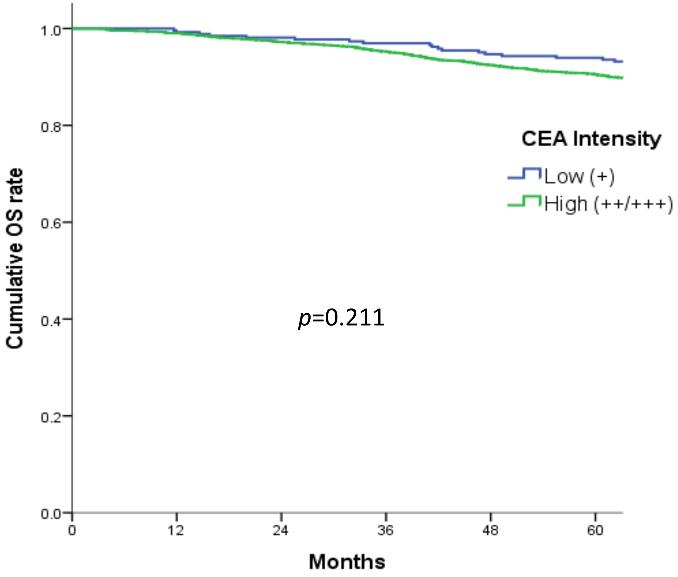

Number at risk:

Low (+)	264	262	259	256	250	248
High (++/+++)	2,121	2,099	2,061	2,020	1,960	1,919
