# Supplementary material for: Clinical implication of tissue carcinoembryonic antigen expression in association with serum carcinoembryonic antigen in colorectal cancer
Source: Sci Rep. 2023 May 10;13:7616. doi: 10.1038/s41598-023-34855-9 (PMC10172318; doi:10.1038/s41598-023-34855-9)
Supplement: Supplementary file 3 — Supplementary Figure 3. [file 41598_2023_34855_MOESM3_ESM.pdf]

Stage III

A

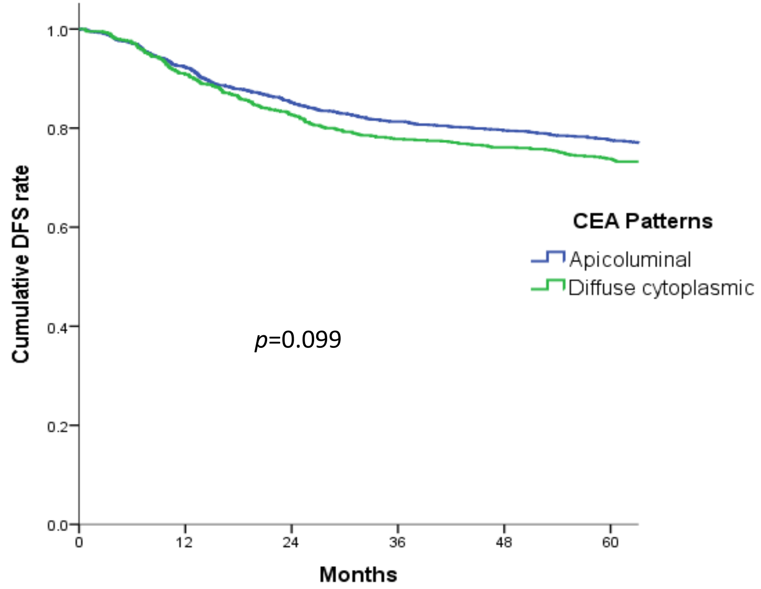

Number at risk:						
Apicoluminal	1,441	1,328	1,226	1,171	1,145	1,117
Diffuse cytoplasmic	829	753	685	645	631	612

B

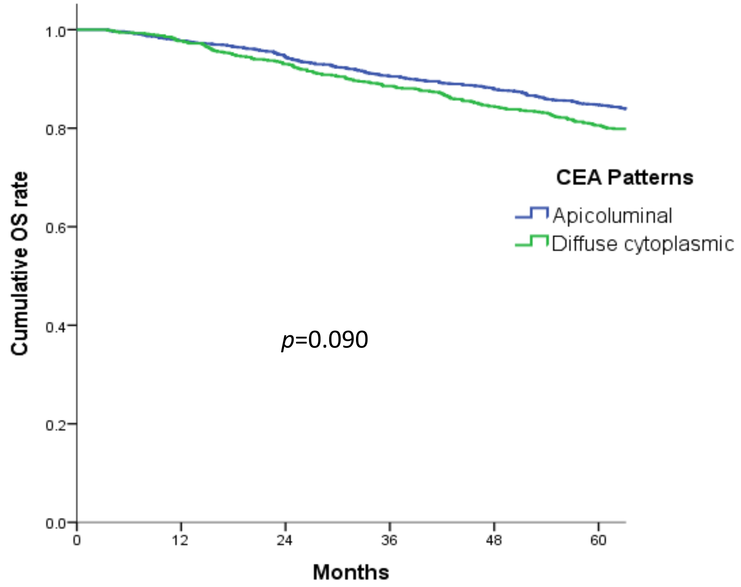

Number at risk:						
Apicoluminal	1,441	1,409	1,361	1,304	1,267	1,221
Diffuse cytoplasmic	829	810	771	734	700	668

C

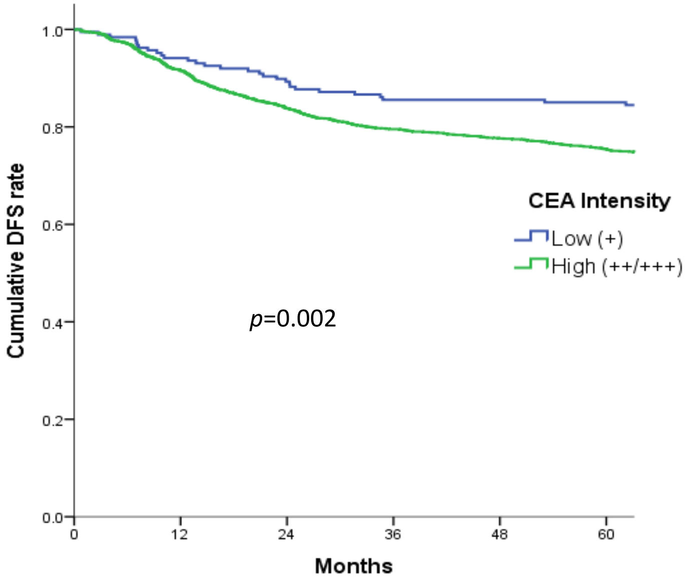

Number at risk:						
Low (+)	187	176	167	160	160	159
High (++/+++)	2,083	1,905	1,745	1,656	1,616	1,570

D

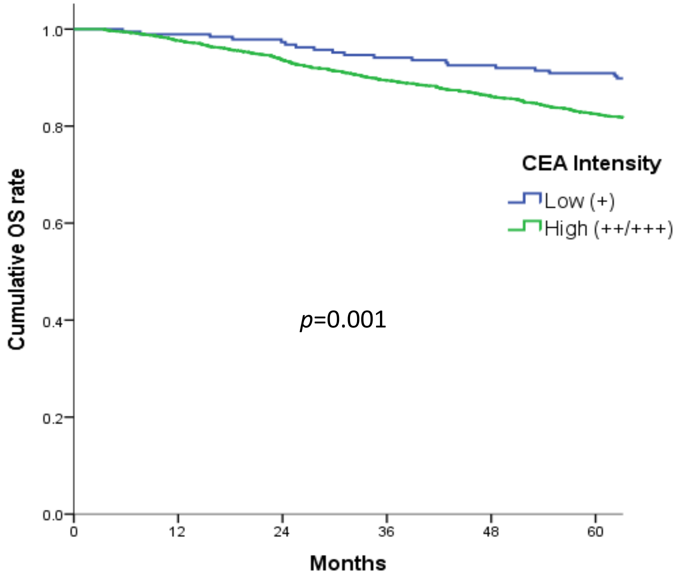

Number at risk:						
Low (+)	187	185	182	176	173	170
High (++/+++)	2,083	2,034	1,950	1,862	1,794	1,719
