# Supplementary material for: Clinical implication of tissue carcinoembryonic antigen expression in association with serum carcinoembryonic antigen in colorectal cancer
Source: Sci Rep. 2023 May 10;13:7616. doi: 10.1038/s41598-023-34855-9 (PMC10172318; doi:10.1038/s41598-023-34855-9)
Supplement: Supplementary file 4 — Supplementary Figure 4. [file 41598_2023_34855_MOESM4_ESM.pdf]

Stage IV

A

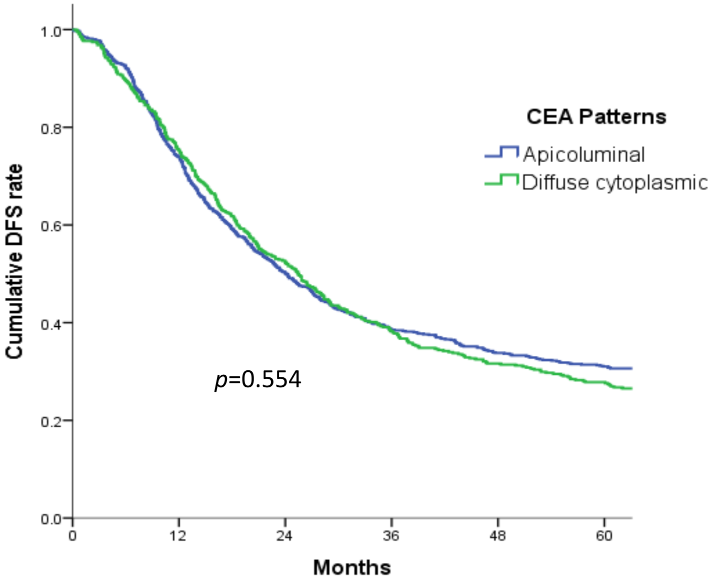

Number at risk:

Apicoluminal	760	562	381	292	256	235
Diffuse cytoplasmic	471	355	247	180	149	131

B

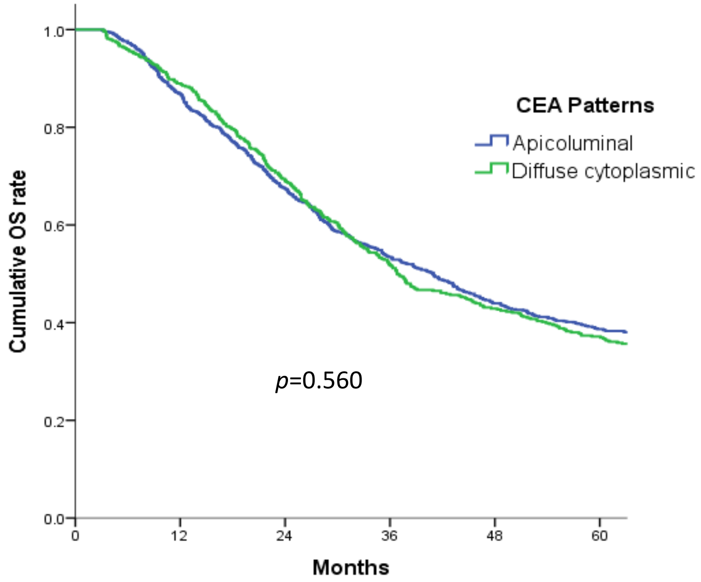

Number at risk:

Apicoluminal	760	660	512	406	334	294
Diffuse cytoplasmic	471	419	326	244	202	175

C

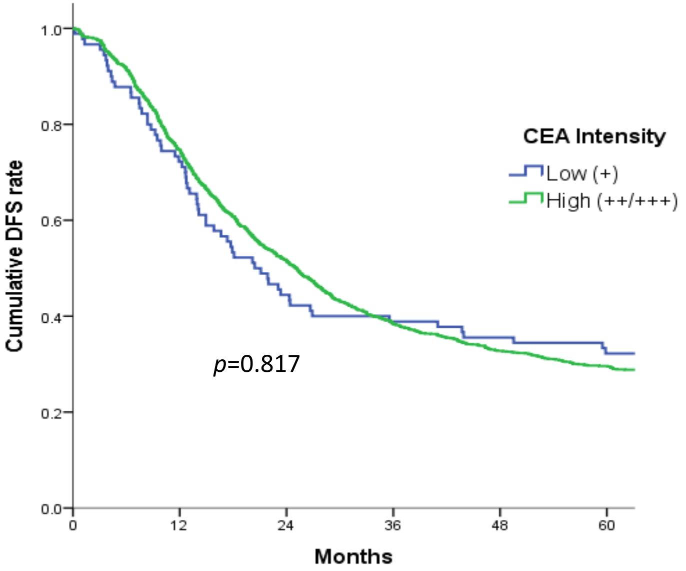

Number at risk:

Low (+)	90	65	40	35	32	29
High (++/+++)	1,141	852	589	437	373	337

D

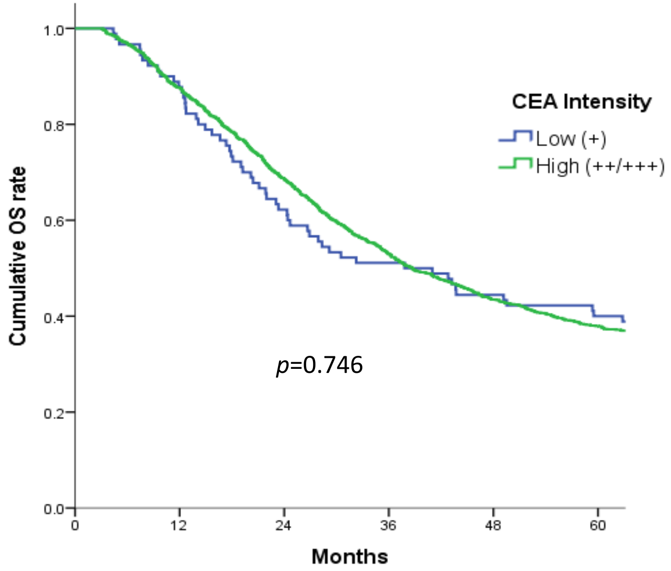

Number at risk:

Low (+)	90	79	56	46	40	36
High (++/+++)	1,141	1,000	783	604	496	433
