# Supplementary material for: Clinical implication of tissue carcinoembryonic antigen expression in association with serum carcinoembryonic antigen in colorectal cancer
Source: Sci Rep. 2023 May 10;13:7616. doi: 10.1038/s41598-023-34855-9 (PMC10172318; doi:10.1038/s41598-023-34855-9)
Supplement: Supplementary file 5 — Supplementary Legends. [file 41598_2023_34855_MOESM5_ESM.docx]

Supplementary Figure 1. Kaplan–Meier analyses comparing (A,C) disease-free survival and (B,D) overall survival in AJCC Stage 0~1 patients with (A,B) the apicoluminal and diffuse-cytoplasmic t-CEA expression patterns, and (C,D) low and high-intensity t-CEA expression.

Supplementary Figure 2. Kaplan–Meier analyses comparing (A,C) disease-free survival and (B,D) overall survival in AJCC Stage 2 patients with (A,B) the apicoluminal and diffuse-cytoplasmic t-CEA expression patterns, and (C,D) low and high-intensity t-CEA expression.

Supplementary Figure 3. Kaplan–Meier analyses comparing (A,C) disease-free survival and (B,D) overall survival in AJCC Stage 3 patients with (A,B) the apicoluminal and diffuse-cytoplasmic t-CEA expression patterns, and (C,D) low and high-intensity t-CEA expression.

Supplementary Figure 4. Kaplan–Meier analyses comparing (A,C) disease-free survival and (B,D) overall survival in AJCC Stage 4 patients with (A,B) the apicoluminal and diffuse-cytoplasmic t-CEA expression patterns, and (C,D) low and high-intensity t-CEA expression.
